# Retinal Vascular Tortuosity in a Patient with Weill-Marchesani Syndrome

**DOI:** 10.1155/2011/952543

**Published:** 2011-12-20

**Authors:** Kevin Gallagher, Tahrina Salam, Barron Sin, Sandy Gupta, Hadi Zambarakji

**Affiliations:** ^1^Department of Ophthalmology, Whipps Cross University Hospital NHS Trust, Whipps Cross Road, Leytonstone, London E11 1NR, UK; ^2^Department of Cardiology, Whipps Cross University Hospital NHS Trust, Whipps Cross Road, Leytonstone, London E11 1NR, UK

## Abstract

Weill-Marchesani syndrome (WMS) is a rare connective tissue disorder with characteristic phenotypic skeletal and ocular manifestations. A 28-year-old myopic female presented with an 8-month history of bilateral blurred vision. On examination, she was noted to be of short stature with brachydactyly. On ocular examination, she was found to be spherophakic with bilateral inferiorly subluxated lenses. Serum and urine homocysteine were normal and a syphilis screen was negative. A diagnosis of Weill-Marchesani syndrome was made. Fundoscopy revealed bilateral tortuous retinal vessels. We report the first illustrated case of retinal vascular tortuosity as an ocular manifestation of Weill-Marchesani syndrome.

## 1. Introduction

Weill-Marchesani syndrome (WMS) is a rare connective tissue disorder with characteristic phenotypic features of short stature, brachycephaly, brachydactyly with limited range of joint movement. Ocular manifestations include microspherophakia with secondary myopia, lens subluxation, and glaucoma secondary to progressive shallowing of the anterior chamber. A review of 128 reported cases of WMS did not describe retinal vascular tortuosity as a feature of the condition [1]. A case series of patients with congenital heart disease identified one patient with WMS with retinal vascular tortuosity (RVT) [2]. We present an illustrated case of retinal vascular tortuosity in WMS in the absence of congenital heart disease.

## 2. Case Report

**Figure 1:**
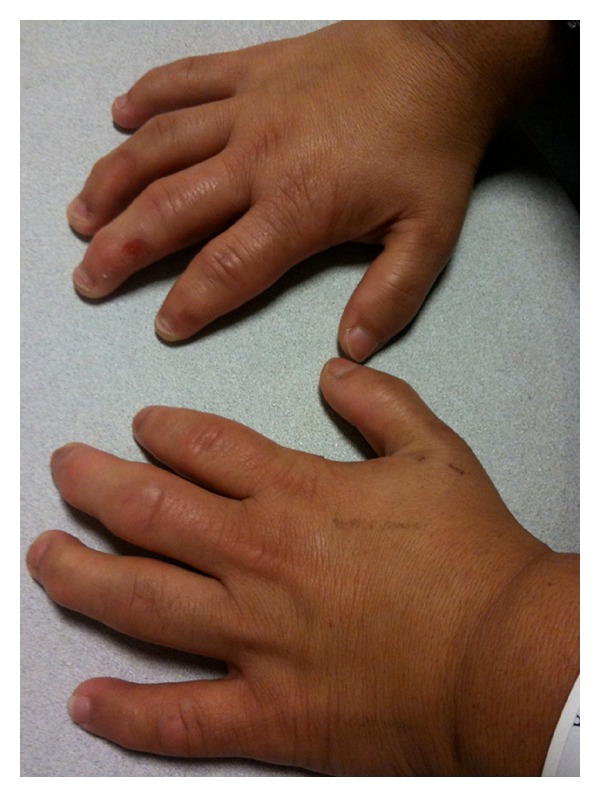
Brachydactyly. The patient had limited joint mobility.

**Figure 2:**
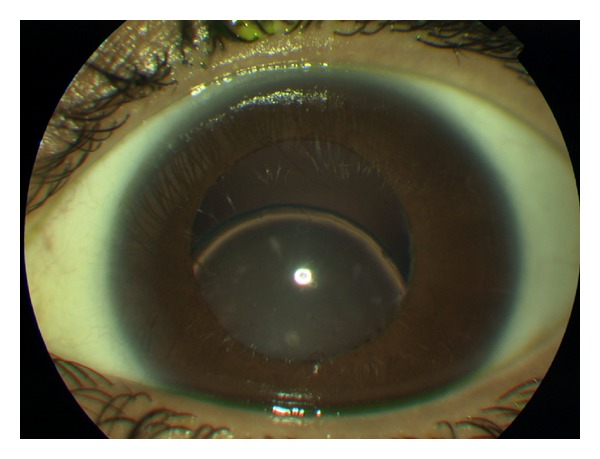
Inferiorly subluxated lens.

**Figure 3:**
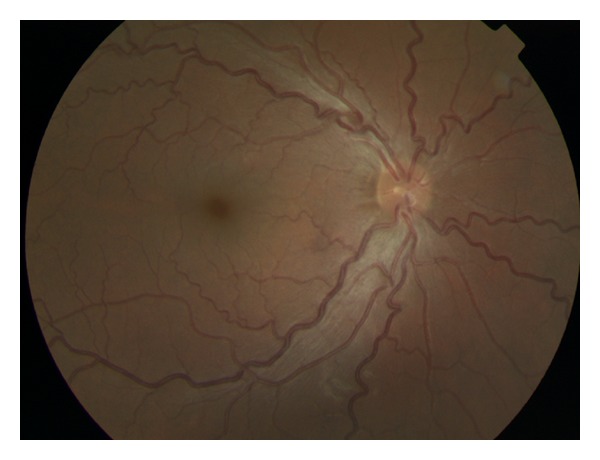
Retinal vascular tortuosity with normal optic discs.

A 28-year-old female presented with an 8-month history of bilateral blurred vision and progressive myopia. Her parents were first cousins. She had three male siblings, all of whom were healthy with no systemic illnesses. She had no other medical history of note. There was no history of ocular trauma. On examination, she was noted to be of short stature with brachydactyly (Figure 1), broad hands and feet, and limited joint mobility. Visual acuity was 0.9log⁡⁡MAR in the right eye and 1.0log⁡⁡MAR in the left. Her most up-to-date refraction was as follows: right −9.25/−3.00 × 160, left −8.75/−4.00 × 40. She was found to be spherophakic with bilateral inferiorly subluxated lenses (Figure 2). Intraocular pressures were 17 and 18 mmHg right and left. Fundoscopy revealed bilateral tortuous retinal vessels with normal optic discs (Figure 3).

Corneal topography was consistent with keratoconus. Keratometry readings on IOL Master (IOL Master v5, Carl Zeiss Meditech) were right K1 47.01 D K2 49.49 D and left K1 54.00 D and K2 63.08 D. Laboratory investigations revealed normal serum and urine homocysteine and a negative syphilis serology.

 The diagnosis of WMS is a clinical one and was made on the basis of short stature and brachydactyly, microspherophakia, ectopia lentis, and the absence of other causes of lens dislocation. While the diagnosis is a clinical one, genetic tests can help confirm the diagnosis. Mutations in the fibrillin-1 gene and the ADAMTS10 gene have been found in autosomal dominant and autosomal recessive forms of WMS [3, 4]. Mutations in the ADAMTS17 gene can give rise to a phenotype resembling WMS [5]. Genetic testing was not performed in this case.

A preoperative cardiology review was requested. Transthoracic echocardiogram has demonstrated normal gross cardiac morphology and good biventricular functions. Mild mitral regurgitation was noted with no haemodynamic consequence.

The patient underwent a pars plana vitrectomy with lensectomy and Artisan anterior chamber intraocular lens (Abbot Medical Optics, Ill, USA) implantation bilaterally. Postoperative visual acuity was 0.24log⁡⁡MAR right and 0.30log⁡⁡MAR left.

## 3. Discussion

We report the first illustrated case of retinal vascular tortuosity associated with WMS, in the absence of congenital heart disease. The association of RVT with WMS has been described in a case series of 240 patients with cyanotic, obstructive, or volume-overloading congenital heart disease [2]. In this series, RVT was strongly correlated with hypoxia and a low haematocrit. Other series of patients with cyanotic congenital heart disease have found an association between RVT and polycythaemia [6, 7]. This finding is supported by the observation that RVT improves with the correction of the cardiac defect and subsequent normalisation of the haematocrit [6]. The patient in this report had no echocardiographic indication of cyanotic, obstructive, or volume-overloading congenital heart disease, was not hypoxic, and had a normal haematocrit. Thus, the retinal vascular changes observed in this case of WMS are likely to represent a different clinical entity to that associated with cyanotic congenital heart disease.
